# Factors associated with mortality risk for malignant colonic obstruction in elderly patients

**DOI:** 10.1186/1471-230X-14-76

**Published:** 2014-04-15

**Authors:** Ming-gao Guo, Yi Feng, Jia-zhe Liu, Qi Zheng, Jian-zhong Di, Yu Wang, You-ben Fan, Xin-Yu Huang

**Affiliations:** 1 Department of Surgery, Shanghai Jiaotong University Affiliated The Six People’s Hospital, 200233 Shanghai, China; 2 Intensive Care Unit, Shanghai Jiaotong University Affiliated First People’s Hospital, 201620 Shanghai, China

## Abstract

**Background:**

Acute colonic obstruction is the most common complication of colorectal cancer (CRC) in elderly patients. Medical treatment has been associated with higher perioperative morbidity and mortality rates. There is a need for identification of elderly CRC patients who will do poorly so that results can be improved. The purpose of this study is to assess the 30-day outcome of elderly patients undergoing malignant colonic obstruction procedures and identify the associated factors of mortality.

**Methods:**

A review of 233 elderly patients who received medical procedures for malignant colonic obstruction between April 2000 and April 2012 was conducted. Data regarding clinical variables, surgical procedures and outcomes, complications, and mortality were studied. Univariate and logistic regression analyses were performed on mortality risk factors.

**Results:**

Patients had a mean age of 78.2 years (range 70–95). A total of 126 (54.1%) patients were classified ASA III and above. Eighty (34.3%) patients had right-sided colonic obstruction. In the 153 (65.7%) patients with left-sided colonic obstruction, 40 patients received self-expandable metallic stent (SEMS) treatment and 193 patients received surgery. A total of 62.2% (n = 145) patients had post operation complications. The overall 30-day mortality was 24.5% (n = 57). ASA grading, peritonitis and Dukes staging were independent risk factors for mortality.

**Conclusions:**

Medical procedures in elderly patients with malignant colonic obstruction are associated with significant complications and mortality. Identifying these high-risk patients and treating promptly may improve outcomes. SEMS treatment provides a useful alternative to surgical intervention.

## Background

Acute colonic obstruction is the most common complication of colorectal cancer (CRC) in elderly patients. The incidence of complete obstruction has been reported to be as high as 30%, and this complication can result in perforation, sepsis, and even death [1-3]. Surgery has been the only therapeutic treatment for this condition for many years. However in the last 20 years, self-expanding metal stents (SEMS) have provided an effective and safe alternative to emergent surgery [4]. Elderly patients often have multiple concomitant illnesses and are usually also in poor clinical condition due to poor physiological reserve and the additional stress caused by the bowel obstruction. Medical treatment has been associated with higher perioperative morbidity and mortality rates in spite of recent improvements in surgical techniques and intensive care. There is an urgent need for identification of elderly patients who will do poorly so that clinical outcomes for this group can be improved.

The purpose of this study is to assess the 30-day outcomes of elderly patients undergoing malignant colonic obstruction procedures and, more importantly, to identify the associated risk factors for mortality in this complex group of patients.

## Methods

From April 2000 to April 2012, we studied retrospective data of consecutive elderly patients (aged ≥70 years) who were diagnosed with acute malignant colonic obstruction and were admitted to the General Surgery Department on an emergent basis. All patients had histologically proven intrinsic colorectal adenocarcinoma. 21 patients with metastatic CRC were excluded. After attempting conservative management with nasogastric suction and intravenous fluid replacement for 24–48 h, 233 patients failed to respond medical management and underwent either SEMS treatment or surgery. Of the 233 patients, 40 with acute left-sided colonic obstruction successfully received SEMS treatment. Placement of SEMS was performed as described previously [4]. Resection of the tumors was attempted in the remaining 193 patients, except in cases of fixed and unresectable tumors or in patients who were hemodynamically unstable. Operative patients who did not undergo resection of the tumors were treated palliatively with stoma or by-pass procedures.

Data regarding the patient’s clinical features, treatment outcomes including 30-day morbidity and mortality, and follow-up information were obtained from a clinical database. We then evaluated clinical factors that could be associated with mortality. The variables included the followinig: age, sex, concurrent illness, time between onset of symptoms and surgery, American Society of Anesthesiologists (ASA) grading, location of obstruction, type of treatment, operative findings (peritonitis, perforations), Dukes staging, and major morbidities. Diagnosis of peritonitis is based upon a clinical history compatible with peritonitis, and an exam in which the abdomen is tender to touch. In addition to pain, the abdomen is rigid and bowel sounds are absent. Mortality was defined as that occurring within 30 days postoperatively.

### Ethical considerations

All treatment interventions applied in this study including SEMS are officially approved, and are routine therapeutic options for old patients in China. Accordingly, prior to the initiation of the treatments, this work was approved by ethics committee at Shanghai Jiaotong University Affiliated The Six People’s Hospital.

### Statistical analysis

Statistical analyses were performed using SPSS software (v.17.0, SPSS). Univariate analysis was performed with the chi-square test. Mann–Whitney U test and Fisher exact test were applied where required. Covariates that remained significant through univariate analysis were selected for multivariate analysis. Forward stepwise logistic regression with P < 0.1 for inclusion and P > 0.05 for exclusion was performed to evaluate mortality. The results were evaluated at a confidence interval of 95%, and significance was set at P < 0.05.

## Results

There were 233 patients in this study with a mean age of 78.2 years (range 70–95). Patient characteristics including gender, time from onset of symptoms to surgery, and ASA grading are shown in Table 1. Comorbidities were identified in 163 (70.0%) patients. Twenty-four (10.3%) patients had two or more comorbidities. The median interval from onset of symptoms to hospital surgery was 54 hours (range 24 –216 h).

**Table 1:** Characteristics of elderly patients who presented with malignant colonic obstruction

	**Patient (n = 233)**	**%**
Gender		
Male	106	45.5
Female	127	54.5
Time from onset of symptoms to surgery, h		
< 72	140	60.1
≥ 72	93	39.9
ASA grading		
I–II	107	45.9
III	60	25.8
IV	61	26.2
V	5	2.1

Surgical observations are shown in Table 2. Eighty (34.3%) patients had right-sided colonic obstruction. Right hemicolectomy was performed in 67 (83.8%) of these patients and ileo-sigmoid bypass was performed in 5 (6.2%) patients. Eight (10.0%) patients with unresectable tumors or metastatic disease received defunctioning loop colostomy.

**Table 2:** Surgical observations, procedures types, and tumor characteristics

**Variables**	**Patient (n = 233)**	**%**
Intra-operative findings		
Peritonitis	46	19.7
Perforations	21	9.0
Location of obstruction		
Right-sided obstruction	80	34.3
Cecum	17	7.3
Ascending colon	20	8.6
Hepatic flexure	23	9.9
Transverse colon	20	8.6
Left-sided obstruction	153	65.7
Splenic flexure	14	6.0
Descending colon	23	9.9
Sigmoid colon	72	30.9
Rectosigmoid	23	9.9
Rectum	19	8.2
Treatment of right-sided obstruction		
Resection + anastomosis	67	83.8
Palliative stoma	8	10.0
Bypass procedure	5	6.2
Treatment of left-sided obstruction		
Surgery	113	73.9
Resection + anastomosis	49	43.4
Resection + stoma	44	38.9
Stoma	20	17.7
SEMS	40	26.1
Dukes staging		
Dukes A and Dukes B	49	21.1
Dukes C	131	56.2
Dukes D	53	22.7

One hundred fifty-three (65.7%) patients presented with left-sided colonic obstruction. Forty (26.1%) of these patients successfully received SEMS treatment. Following SEMS insertion, staging and systemic evaluation of fitness for operation was performed. Seven of the 40 patients (17.5%) were considered ineligible for surgery because of serious comorbidities, and in these patients, SEMS was used as permanent palliative therapy. The remaining 33 of the 40 (82.5%) patients successfully received SEMS as a bridge to surgery. Tumor resection and primary anastomosis were performed in 30 (75.0%) patients, and Hartmann’s procedure was performed in 3 (7.5%) patients. Surgery was performed in 113 (73.9%) patients. RPA (resection + primary anastomosis) was performed in 49 (43.4%) patients and RS (resection + stoma) was performed in 44 (38.9%) patients. Twenty (17.7%) patients received palliative colostomy procedures.

A total of 62.2% (n = 145) patients had post operation complications of some kind. Local and systemic complications occurred in 15.5% (n = 36) and 46.8% (n = 109) respectively (Table 3). Surgical site infection (9.9%) was the leading complications caused by surgery. Respiratory and cardiac complications (15.5% vs 10.3%) were the main post-operative morbidities in these elderly patients with malignant colonic obstruction.

**Table 3:** Main post-operative complication

**Complication**	**Patient (n = 233)**	**%**
Surgical		
Surgical site infection	23	9.9
Anastomotic leak	3	1.3
Intestinal obstruction	7	3.0
Wound dehiscence	3	1.3
Medical		
Respiratory complications*	36	15.5
Cardiac complications^&^	24	10.3
Cerebrovascular complications	4	1.7
Urinary tract complications	9	3.9
Renal complications	23	9.9
Thromboembolism	5	2.1
Multiorgan failure	8	3.4

*Respiratory complications include ARDS, pneumonia, suffocation.

&Cardiac complications include cardiac arrhythmia, myocardial ischemia, myocardial infarction, cardiac failure, pulmonary edema.

The overall 30-day mortality was 24.5% (n = 57). In left-sided colonic obstruction, a lower mortality (7.5%) was found in patients who received SEMS compared to patients who received surgical treatment (28.3%) (p < 0.01). Univariate analysis of risk factors for mortality showed that time from onset of symptoms to surgery, ASA grading, intra-operative findings, Dukes staging, and systemic morbidity reached statistical significance (Table 4). Multivariate analysis indicated ASA grading and peritonitis were independent risk factors for mortality (Table 5). In addition, Dukes staging with an OR of 2.55 (95% CI: 1.03-3.39, P = 0.04) was another risk factor.

**Table 4:** Univariate analysis of 30-day mortality for a series of elderly patients who underwent treatment for malignant colonic obstruction

**Variables**	**Deaths/patients (%)**	**P value**
Mortality	57/233 (24.5)	
Age		NS
70-79	28/138 (20.3)	
≥ 80	29/95 (30.5)	
Sex		NS
Male	27/106 (25.5)	
Female	30/127 (23.6)	
Comorbidities		<0.01
Negative	8/70 (11.4)	
Positive	49/163 (30.1)	
Time from onset of symptoms to surgery, h		<0.01
< 72	24/140 (17.1)	
≥ 72	33/93 (35.5)	
ASA grading		<0.01
I–II	5/107 (4.7)	
III	15/60 (25.0)	
IV	32/61 (52.5)	
V	5/5 (100.0)	
Intra-operative findings		
Peritonitis	25/46 (54.3)	<0.01
Perforations	14/21 (66.7)	<0.01
Location of obstruction		NS
Right-sided obstruction	25/80 (31.3)	
Left-sided obstruction	32/153 (20.9)	
Dukes staging		<0.01
Dukes A and Dukes B	5/49 (10.2)	
Dukes C	31/131 (23.7)	
Dukes D	21/53 (39.6)	
Morbidities		
Surgical	12/36 (33.3)	NS
Medical	45/109 (41.3)	<0.01

NS no significance.

**Table 5:** Multivariate analysis of mortality for a series of elderly patients who underwent treatment for malignant colonic obstruction

	**Odds ratio (95**% **CI)**	**P value**
ASA grading		
I–II	Reference	Reference
III ,IV,V	4.85 (2.95-7.99)	<0.01
Peritonitis		
No Peritonitis	Reference	Reference
Peritonitis	3.89 (1.41-9.35)	<0.01
Dukes staging		
Dukes A and B	Reference	Reference
Dukes C and D	1.87 (1.03-3.39)	0.04

## Discussion

Colorectal cancer is predominantly a disease of elderly people and is a major cause of morbidity and mortality in the elderly population [5]. Analysis of published reports shows that elective CRC resection in the elderly population is worthwhile and should be carried out for the same indications as for younger patients [6,7]. Acute mechanical bowel obstruction is more frequent in elderly patients, and studies have shown that up to 30% of these patients present with this complication [1-3,8].

Surgery for malignant colorectal obstruction poses several problems. Elderly patients often have an increased incidence of comorbid conditions. However, in subgroups of aged patients undergoing surgery, there has been no evidence of increased comorbidity with increased age [5]. Our study confirmed the results of previously published studies in which the incidence of comorbidity ranged from 58.0 to 81.5% [9-12]. Results describing whether underlying chronic conditions are responsible for the increased morbidity and mortality remain discordant. Ozkan reported that comorbid conditions influenced the mortality, while Rubinfeld showed that none of the comorbidities in his study accurately predicted mortality in aged patients who received an emergency major abdominal operation [12,13]. Our study showed that comorbidity was not a significant prognostic factor for aged patients in multivariate analysis.

The placement of a metallic stent in the colon was first reported by in 1991 by Dohmoto [14]. Since then, many reports have suggested a major role for SEMS placement in patients with limited life expectancy due to nonresectable tumors or progressive metastatic disease, with stent placement being very effective in avoiding the need for palliative diverting colostomy. The indications for stent placement have been extended also to patients with resectable colorectal cancer, as a bridge to elective surgery, allowing a more complete preoperative evaluation and more detailed bowel preparation [4,15]. This retrospective study shows placement of SEMS to be an appealing option either for palliation or as a bridge to semi-elective surgery. Compared to emergent surgery, patients treated with SEMS had a significantly lower mortality rate. Among patients who received SEMS, our study showed a higher rate of primary anastomosis (75.0% vs 43.4%) and a marked reduction in stoma formation (7.5% vs 56.6%) when compared with patients who received emergent surgery for left-sided colonic obstruction. SEMS provides a useful alternative to emergent surgical intervention in the management of acute left-sided colorectal obstruction for elderly patients [4].

As in previous reports, for emergent surgery in the unprepared left-sided colon, RS is still one of the best operative alternatives, especially in elderly patients [16-18]. In our research, a higher number of RPA (43.4%) and a lower number of RS (38.9%) were performed in elderly patients. It is important to consider that the choice of procedure was not randomized, rather, RPA was only carried out when the surgeons believed that local and systemic conditions of the aged patients were appropriate. The high rate of stoma formation might be attributed to hemodynamically unstable states and lower physiological reserves in octogenarian patients. RPA was used for 116/233 patients in our study, and 3 (2.6%) of these patients presented with anastomotic dehiscence. Compared to the 3.0% and 6.3% reported in previous series [16,17], the anastomotic dehiscence rate was not higher in elderly patients. No significant difference in morbidity and mortality with regard to RPA vs RS was found. RPA for malignant colonic obstruction should be encouraged when the over-all physical condition of the elderly patient is appropriate.

Management of obstructing colorectal cancer remains strongly associated with poor outcomes. Ozturk reported in a multivariate logistic regression analysis that colorectal disease increased the risk of death by ninefold in elderly patients undergoing surgery [19]. Emergency surgery, a well-known risk factor, increases operative mortality rates between 3-fold and 10-fold. This research confirm the results of previously published studies, in which morbidity rates of 25–81% and mortality rates of 17–30% have been reported [20-23].

The first objective of our study was to identify correlates of poor clinical outcomes as possible risk factors in elderly patients who received treatment of colorectal obstruction. Univariate analysis found that high mortality was associated with time from onset of symptoms to surgery, ASA grading, presence with peritonitis or perforations, Dukes staging and systemic morbidity. Logistic regression analysis was used to test for the possibility of all these collected parameters to predict the poor outcome. As reported by previously published studies, ASA grading was found to be the most important determinant of mortality [12,20]. Physiological and Operative Severity Score for the enUmeration of Mortality and Morbidity (POSSUM) is also a valuable and effective scoring system for calculating operative risk [24]. But it includes preoperative laboratory findings, which makes it harder to use, the same problem as seen with the Acute Physiology and Chronic Health Evaluation (APACHE) [5]. Our study shows peritonitis to be a more important prognostic factor than time from onset of symptoms to surgery in emergent colonic obstruction surgery, a result consistent with previous study [16]. Dukes staging was a risk factor associated with death. This result may be attributed to the poorer physiological reserve associated with later stage disease in elderly patients.

The term ‘getting old’ may be defined as ‘an inherent, progressive impairment of function with the passage of time, which cannot be averted, and which causes individuals to become more vulnerable to death’ [25]. But it is difficult to define an age limit. As indicated in most previous studies regarding this definition, the physiological and chronic health status of the patient are much more important than the chronological age. No significant correlation between age and poor clinical outcome was found in this study.

## Conclusions

In order to prevent high mortality, it should be emphasized that in elderly patients who require operation for colorectal cancer, the operation should be performed as soon as conditions are optimized and should not be postponed. When malignant colonic obstruction occurs, identifying these high-risk patients and treating them promptly rather than delaying or denying surgery is the best option. Also SEMS may provide a useful alternative to surgical intervention in the management of acute left-sided colorectal obstruction for elderly patients.

## Competing interests

The authors declare that they have no competing interests.

## Authors’ contributions

MG: concept, design, data analysis, manuscript writing and editing. YF: concept, design, data analysis, manuscript writing. JL: concept, design, data analysis, manuscript writing and editing. QZ: concept, design, manuscript editing. JD: statistical analysis. YW: design of the study. YF: manuscript editing. XYH: concept, design. All authors read and approved the version to be published.

## Pre-publication history

The pre-publication history for this paper can be accessed here:

http://www.biomedcentral.com/1471-230X/14/76/prepub
